# Exercise as adjunctive treatment for alcohol use disorder: A randomized controlled trial

**DOI:** 10.1371/journal.pone.0186076

**Published:** 2017-10-19

**Authors:** Kirsten K. Roessler, Randi Bilberg, Anette Søgaard Nielsen, Kurt Jensen, Claus Thorn Ekstrøm, Sengül Sari

**Affiliations:** 1 Department of Psychology, University of Southern Denmark, Odense M, Denmark; 2 Unit of Clinical Alcohol Research, Clinical Institute, University of Southern Denmark, Odense C, Denmark; 3 Department of Sports Science and Clinical Biomechanics, University of Southern Denmark, Odense M, Denmark; 4 Department of Biostatistics, University of Copenhagen, Copenhagen K, Denmark; Vanderbilt University, UNITED STATES

## Abstract

**Aims:**

To examine whether physical activity as an adjunct to outpatient alcohol treatment has an effect on alcohol consumption following participation in an exercise intervention of six months’ duration, and at 12 months after treatment initiation.

**Methods:**

The study is a randomized controlled study with three arms: Patients allocated to (A) treatment as usual, (B) treatment as usual and supervised group exercise, (C) treatment as usual and individual physical exercise. The primary outcome measure was excessive drinking six months after treatment start and completion of the intervention. A logistic regression model was used to evaluate the odds of excessive drinking among the three groups, based on intention-to-treat. Changes in level of physical activity in all three groups were tested by using a generalized linear mixed model. A multiple linear model was used to test if there was an association between amount of performed physical activity and alcohol consumption.

**Results:**

A total of 175 patients (68.6% male) participated. Response rates were 77.7% at six months and 57.1% at 12 months follow-up. OR 0.99 [95% CI: 0.46; 2.14], p = 0.976 for excessive drinking in the group exercise condition, and 1.02 [95% CI: 0.47; 2.18], p = 0.968 in the individual exercise condition, which, when compared to the control group as reference, did not differ statistically significantly. Participants with moderate level physical activity had lower odds for excessive drinking OR = 0.12 [0.05; 0.31], p<0.001 than participants with low level physical activity. Amount of alcohol consumption in the intervention groups decreased by 4% [95% CI: 0.03; 6.8], p = 0.015 for each increased exercising day.

**Conclusions:**

No direct effect of physical exercise on drinking outcome was found. Moderate level physical activity was protective against excessive drinking following treatment. A dose-response effect of exercise on drinking outcome supports the need for implementing physically active lifestyles for patients in treatment for alcohol use disorder.

## Introduction

Treatment of alcohol use disorder (AUD) is associated with a range of challenges, and meta-analyses suggest the effect size to be low to moderate [1]. Aspects associated with negative outcome of treatment include having less social stability and support, lacking a social network of non-drinkers, a family history of alcohol dependence, psychiatric comorbidity, multiple previous treatment episodes, a history of disengagement from treatment, cognitive deficit, low alliance, personality disorder, and young age [2–4]. Therefore, specialized treatment of AUD may benefit from some improvement strategies. Especially in the later stages of treatment, focusing on reintegration into society and restoration of normal function, including establishing a healthy lifestyle is important [2]. Innovative interventions such as physical exercise as add-on treatment to current treatment are therefore suggested to have promising effects [5–8].

Clinical evidence from studies of exercise in smoking cessation and alcohol abuse suggest that exercise may directly impact stimulant use and mediate withdrawal symptoms [9]. Also, in treatment of chronic disorders, exercise appears to mediate important health related outcomes such as mood, quality of life, sleep, and cognitive function [9]. Since people with AUD in general tend to have worse physical health, including a higher prevalence of metabolic syndrome, cardiovascular diseases, diabetes, certain cancers, and also lower physical fitness compared to the rest of the population [10, 11], an increased attention on physical exercise among this patient group would appear to be relevant. In addition, a recent meta-analysis of exercise interventions for alcohol dependence showed significant positive effects on fitness and depression [12]. Types of intervention included in the meta-analysis were aerobic exercise, a combination of aerobic exercise, strength training and/or calisthenics, and yoga/stretching [12].

Hence, exercise may be a promising intervention for individuals with stimulant use disorders in general, in order to potentially reduce consumption, but also to reduce the comorbid diseases linked specifically to AUD.

Only few studies until now have tested exercise in randomized trials, investigating the effect of adding exercise to treatment of AUD. According to a recent review [8], three out of five randomized studies found that the intervention group demonstrated a greater decrease in craving for alcohol than a comparison group [13], increased abstinence rates and decreased frequency and amount of alcohol use [5]. Two studies [14, 15] found no differences between experimental and comparison groups. However, due to the methodological limitations of these studies, such as small sample sizes and high dropout, a large randomized controlled study is needed. The Healthy Lifestyle Study [16] seeks to meet this need. The Healthy Lifestyle Study is part of a large suite of randomized controlled studies bearing the acronym RESCueH, and aiming to test non-pharmacological outpatient alcohol treatment [17]. All the studies in RESCueH are designed to investigate whether the effectiveness of current outpatient treatment of AUD increases with the inclusion of practical interventions. RESCueH stands for: **R**elay Study, **E**lderly Study, **S**elf-match Study, **Cue** Exposure Study and **H**ealthy Lifestyle Study.

### Objective and hypotheses

The objective of the present study is to examine whether physical activity as an adjunct to outpatient alcohol treatment has an effect on alcohol intake at six months and 12 months after treatment initiation, in particular, the amount and frequency of alcohol intake, including number of heavy drinking days.

We hypothesized that adding physical exercise of moderate intensity to treatment as usual for AUD would yield significant clinical improvements regarding the amount and frequency of alcohol intake, such that exercising patients would have significantly lower consumption than non-exercising patients.

In terms of secondary outcomes, patients’ wellbeing, fitness, anxiety, depression and interpersonal problems were also examined in the study. The secondary findings will be reported elsewhere.

Pre-registered hypothesis: http://www.isrctn.com/ISRCTN74889852

## Materials and methods

### Design

The study was a randomized controlled study with three arms: (A) Patients allocated to treatment as usual, (B) Patients allocated to treatment as usual and supervised group exercise, (C) Patients allocated to treatment as usual and individual physical exercise. The specific forms of exercise (aerobic) were brisk walking and running performed outdoors [16]. Some evidence suggests that exercise performed outdoors has better mood-enhancing effects than indoor activities [18, 19]. Furthermore, running has become very popular in Denmark in recent years [20]. Thus, the rationale for choosing this form of exercise was based on existing evidence. In addition, the length of the intervention was six months, as we aimed to test the long-term effects of the intervention on drinking outcomes. The patients were randomized by an independently performed urn-randomization. All patients were assessed at baseline (before treatment start and at time of enrollment in the study), and at six and 12 months after enrollment in the study. The study was designed to be compliant with the CONSORT statement.

### Interventions

Participants in the *Control group* A were asked not to change their physical activity levels during the intervention period. All participants, including the patients randomized to the control group, were invited to do a cardiorespiratory fitness test at baseline prior to intervention start, and at six and 12 months thereafter.

In group B, *Supervised group exercise* participants received, in addition to TAU, a supervised program and running instructions depending on their level of experience. They exercised for 24 weeks and were asked to meet for the group exercise twice a week during the intervention period. The group was supervised by running instructors with backgrounds in sports science and psychology.

In group C, *Individual exercise*, participants received, in addition to TAU, a written training program with running instructions during an individual session prior to start. This session was followed up once or twice during the intervention period if the participant requested it. During the six-month long intervention period, the participants followed their self-organized individual training program which was designed to encourage them to exercise at least twice a week. The participants in both intervention groups were asked to record their exercise activity using heart rate monitors in the form of watches. The intervention is further described elsewhere [16].

### Setting

The study was conducted in two Danish outpatient treatment centers. In Denmark, there is a legal duty on municipalities to provide treatment for alcohol use disorders free of charge. Typically, the treatment centers offer motivational interviewing, cognitive behavioral therapy and family therapy. Most treatment centers also offer acute treatment for withdrawal symptoms and other forms of pharmacological treatment [21]. The length of treatment is decided in conjunction with the patient on an individual basis with no time limit. However, the national clinical guideline for the treatment of alcohol dependence recommends planning structured alcohol treatment of three months’ duration. Furthermore, patients have the option of remaining anonymous during treatment [21].

### Measures

#### Primary outcome

The primary outcome measure was alcohol intake six months following treatment start after completion of the physical exercise intervention.

#### Data

At all data-collection points, data was collected by the means of three instruments: The Addiction Severity Index, the Timeline Follow-Back questionnaire, and the International Physical Activity Questionnaire.

#### Addiction severity

The Addiction Severity Index (ASI) [22] provides a multidimensional image of the patient’s psychosocial and addiction-related situation within the last month before the interview. The interview concentrates on the following seven areas in the patient’s life: medicine, employment, alcohol use, drug use, legal status, family or social network, and psychiatric health. The ASI features two separate scores: the interviewer’s score and the composite score. The scores give a mathematical estimate of each problem area based on symptoms within the 30-day period preceding the interview. Each composite score consists of the sum of the various items on the ASI. Final scores are reported as continuous values from 0 to 1, where 0 denotes no problems and 1 denotes severe problems. ASI is a very common and valid measure of addiction severity widely used in addiction research, and in the treatment facilities included in the present study [21, 22].

#### Alcohol consumption

The Timeline Follow-Back questionnaire (TLFB) [23] is used to demarcate alcohol-free days from drinking days as well as to measure the number of drinks consumed per day. Using the TLFB questionnaire, patients estimate the number of standard drinks (one unit contains 12 grams of pure alcohol) they consume in the 30 days before treatment initiation and the 30 days before the 6-month and 12-month follow-up interviews. The TLFB is used to yield the average number of drinks consumed per day, the number of any drinking days, and calculates number of heavy drinking days. A heavy drinking day was defined as six or more drinks per day, according to the recommendations of the Danish Health Authority [24].

#### Physical activity

The International Physical Activity Questionnaire (IPAQ) [25] is used to assess physical activity undertaken across a comprehensive set of domains, including leisure-time physical activity, domestic and gardening activities, work-related physical activity, and transport-related physical activity. The short form asks about three specific types of activity undertaken in the four mentioned domains: walking, moderate-intensity activities, and vigorous-intensity activities.

In addition, the exact level of physical exercise of each participant in the individual and in the group condition was measured by heart rate monitors (Polar RC3 GPS with Heart Rate Sensor), which the participants were asked to wear every time they exercised.

#### Ethical approvals

The study is presented to and approved of The Regional Scientific Ethical Committee for Southern Denmark (J.nr.S-20130031) and the Danish Data Protection Agency. All procedures in the study are in accordance with the second Declaration of Helsinki.

### Participants

A total of 175 consecutive participants were recruited from the two outpatient alcohol treatment centers in Odense (n = 148) and Svendborg (n = 27) at the time of initiation of their treatment course for AUD between 1^st^ May 2013 and 19^th^ March 2015. They were 45 years old on average with a range from 21 to 70 (SD = 11.3). The participants have had an excessive use of alcohol for 14.8 years on average (SD = 10.6) and met the following inclusion criteria: fulfilling ICD-10 criteria for harmful use of or dependence on alcohol, age over 18 years, Danish speaking, no severe psychosis or cognitive impairment, no severe physical disabilities or medical problems and acceptance of participation in the study. All participants provided written and oral informed consent.

**Fig 1:**
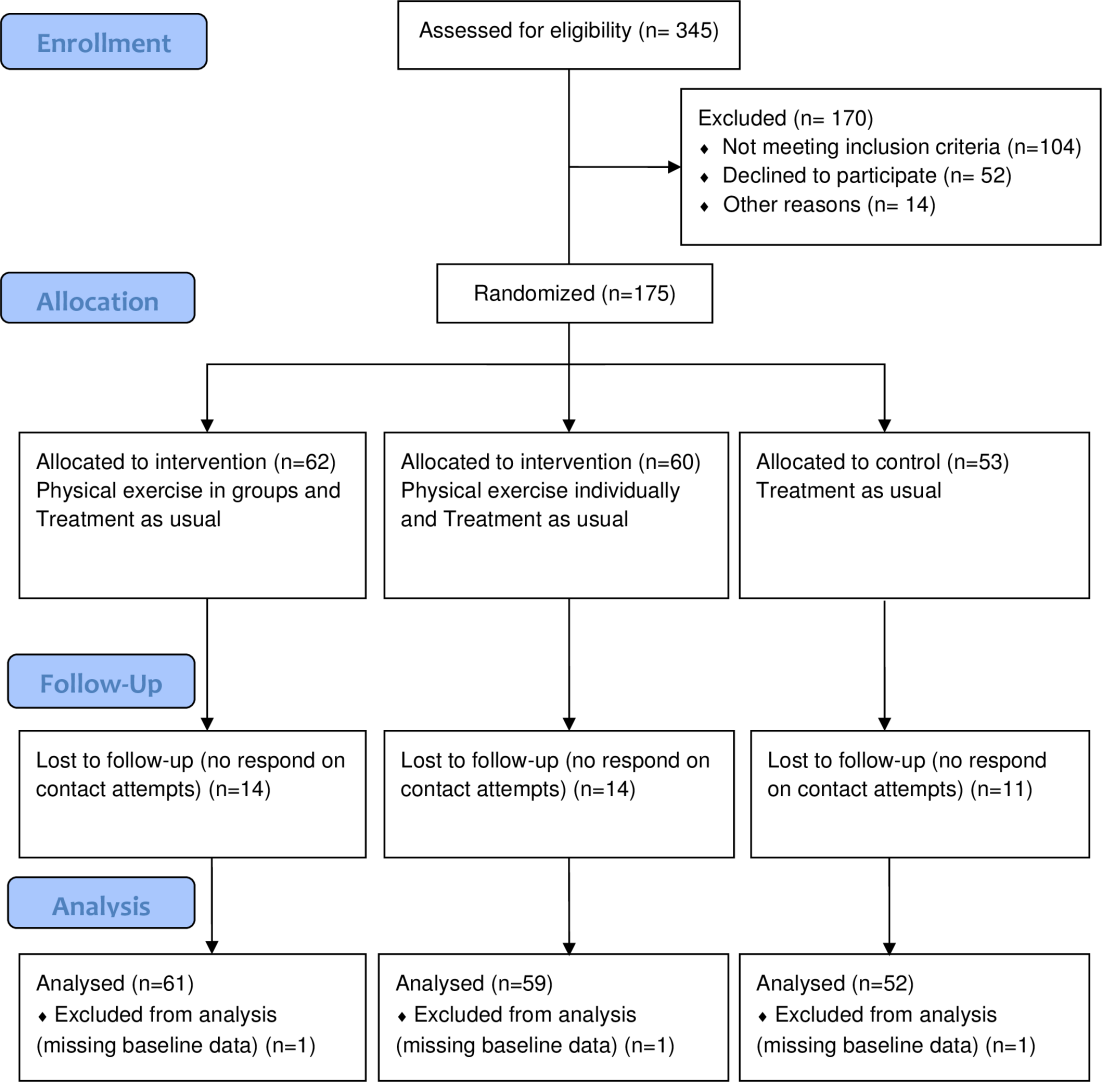
CONSORT flowchart of participant inclusion, follow-up and analysis.

Fig 1 illustrates the patient flow in our study. Of the 175 patients who were eligible to participate, three participants were missing three, four and seven days of data, respectively, in the baseline TLFB administration. For these participants, the missing days were filled in on the TLFB calendar with zero drinks. Three participants did not complete the baseline interview, hence a total of 172 participants are considered study participants. Of these 172 patients, 136 completed the six-month follow-up ASI-interview, hereof 113 patients returned the six-month TLFB questionnaire, and 100 completed the twelve-month follow-up interview.

### Data analysis and statistics

#### Outcome measures

The primary outcome measure was the proportion of patients who did not drink excessively six months after treatment start. Not drinking excessively was defined as being either abstinent or drinking moderately during the last 30 days prior to the follow-up interview. Moderate drinking was defined as drinking a maximum of 14 and 21 standard units of alcohol per week, respectively, for women and men, and a maximum of 5 standard units of alcohol on a drinking day [24], again during the last 30 days prior to follow-up.

Secondary analyses evaluated abstinence during the last 30 days prior to the follow-up six months after treatment start, and alcohol consumption on drinking days during the month prior to follow-up.

### Analysis

Descriptive analyses of the participants’ characteristics were conducted using summary statistics. The baseline data are shown in Table 1, where the variables are summarized as mean and standard deviation (SD) or median and inter-quartile range for symmetric/bell-shaped and non-normally distributed numeric variables, respectively. Categorical variables are presented as frequencies and relative frequencies for categorical variables. For the 6 month clinical characteristics, unadjusted comparisons between the three groups were based on one-way ANOVA or Kruskal-Wallis test, when appropriate (Table 2).

**Table 1:** Baseline demographics and clinical characteristics of the study sample (N = 175).

	Group exercise	Individual exercise	Control
	n = 62	n = 60	n = 53
Age mean (SD)	44.8 (11.2)	43.8 (11.1)	46.9 (11.6)
Male n (%)	36 (59.0)	45 (77.6)	39 (73.6)
Education n (%)			
None	19 (31.7)	16 (28.1)	12 (23.1)
Technical	25 (41.7)	26 (45.6)	25 (48.1)
College	16 (26.7)	15 (26.3)	15 (28.8)
Employed n (%)	31 (51.7)	37 (64.9)	28 (54.9)
Marital status n (%)			
With partner	19 (31.7)	12 (21.1)	27 (51.9)
Without partner	41 (68.3)	45 (78.9)	25 (48.1)
Alcohol use^a^ median [iqr]	145.0 [69.0, 310.0]	281.0 [157.2, 442.5]	210.9 [62.0, 327.0]
Excessive drinking^b^	53 (86.9)	55 (94.8)	45 (84.9)
PDD^c^ [iqr]	50 [23.3, 80.0]	71.67 [46.46, 90.0]	73.33 [30.0, 90.0]
DDD^d^	11.4 [7.6, 18.4]	15.1 [10.0, 21.3]	11.4 [8.5, 15.1]
ASI score^e^	0.67	0.71	0.70

Notes

^a^Alcohol units consumed 30 days prior to treatment start

^b^ >14 (women) and 21 (men) standard units of alcohol per week, or >5 standard units of alcohol per day

^c^PDD: Percent drinking days

^d^DDD: Drinks per drinking day

^e^ Addiction Severity Index 0 = no problem, 1 = severe problem.

**Table 2:** Six month clinical characteristics of the study sample (N = 113).

	Group n = 40	Individual n = 36	Control n = 37	p-value
Alcohol use^a^ median[iqr]	0 [0, 57]	0 [0.00, 30.5]	0 [0, 36.0]	0.757
Excessive drinking^b^	15 (37.5)	10 (27.8)	14 (37.8)	0.588
PDD^c^ [iqr]	0.0 [0.0, 14]	0.0 [0.0, 20]	0.0 [0.0, 16.7]	0.888
DDD^d^ [iqr]	8.6 [5.0, 15.4]	6.0 [3.0, 13.5]	13.5 [4.4, 21.2]	0.340

Notes

^a^Alcohol units consumed 30 days prior to treatment start

^b^ >14 (women) and 21 (men) standard units of alcohol per week, or >5 standard units of alcohol per day

^c^PDD: Percent drinking days

^d^DDD: Drinks per drinking day.

#### Primary outcome

A logistic regression model was used to evaluate the odds of excessive drinking among the three treatment groups [26]. Analysis of outcome was based on intention-to-treat, irrespective of whether the patients had dropped out of the intervention. If data were missing at the six months follow-up, baseline data was used (essentially assuming that non-responders had relapsed) which results in conservative estimates of the treatment effect and potentially upwardly-biased significance tests (i.e. too large p-values). The dropout rate was identical for all three treatment groups. Secondary intention-to-treat outcomes such as proportion of abstinence and drinks per drinking day at six months were analyzed similarly. We also considered the number of standard units of alcohol assessed at baseline and at both follow-ups as derived from TLFB for non-abstinent individuals.

In order to take changes in level of physical activity in all three groups during the period under study into consideration, including the control group, we analyzed the IPAQ data for all groups. The physical activity categories *low*, *moderate* and *high* were calculated according to the IPAQ guidelines. Individuals who did not meet the criteria for the categories *moderate* or *high* were considered to have a low physical activity level. The pattern of activity to be classified as *moderate* is five or more days of any combination of walking, moderate-intensity or vigorous-intensity activities achieving a minimum total physical activity of at least 600 MET-minutes/week. The criterion for classification as *high* is seven or more days of any combination of walking, moderate-intensity or vigorous-intensity activities achieving a minimum total physical activity of at least 3000 MET-minutes/week [27]. For the IPAQ analysis we considered the full span of follow-up (both six months and 12 months) and used a generalized linear mixed model appropriate for repeated registrations of excessive drinking. The generalized linear mixed model was used to model the odds of excessive drinking as a function of treatment group and period. The same model was also used to model the number of drinking days and drinks per drinking day in the intervention groups relative to the control group when analyzing the IPAQ data. We included an interaction between treatment groups and time periods to allow the curves for the three treatments to vary among treatment groups.

#### Dose-response analysis

Lastly, we conducted a dose-response analysis in order to test if there was an association between the amount of performed physical activity and reduced alcohol consumption. For this analysis, we used a multiple linear model [26] and included the number of running days, measured by the heart rate monitors, as a predictor. Level of statistical significance was determined at p < 0.05 for all analyses.

## Results

### Descriptive results

Table 1 presents characteristics in the treatment and control groups at baseline, and Table 2 presents clinical characteristics of the groups at six months follow-up.

### Primary outcome

At the time of the six months follow-up, all three groups showed a marked reduction in alcohol intake (the controls dropped from 88% excessive drinking to 54%, group exercise dropped from 91% to 54% excessive drinking and individual exercise from 95% to 54%). No differences between the groups were found in the proportion of patients who drank excessively (Table 2). Participants allocated to exercise and participants allocated to the control group were not significantly different from each other in relation to drinking outcomes measured as consumed units of alcohol per month at follow-up. The number of days abstinent had increased while the number of drinks per drinking day had decreased across the total sample.

Table 3 shows the ORs for drinking likelihood in intervention groups compared to the control group measured at six months after treatment initiation. The control group is the reference group. The OR 0.99 for excessive drinking in the group exercise condition, and 1.02 in the individual exercise condition, which, when compared to the control group as reference, did not differ statistically significantly. The relative risk for drinks per drinking day in the individual exercise condition compared to the control group was 0.39, p = 0.059, which is close to statistically significant.

**Table 3:** Effect of exercise on AUD at six months follow-up. Intention-to-treat in relation to control group with last observation carried forward if follow-up data on alcohol intake is incomplete N = 172, df = 1).

	Effect [95% CI]	p-value	Effect [95% CI]	p-value
		(test statistics)		(test statistics)
	Group exercise		Individual exercise	
	(N = 61)		(N = 59)	
Excessive drinking^a^ OR	0.99 [0.46; 2.14]	0.976 (z = -0.03)	1.02 [0.47; 2.18]	0.968 (z = 0.04)
Abstinence rate^b^ OR	1.06 [0.50; 2.28]	0.860 (z = -0.18)	0.94 [0.43; 2.02]	0.868 (z = 0.17)
NDD^c^	-2.68 [-8.48; 3.13]	0.370 (t = -0.90)	-3.60 [-10.04; 2.84]	0.279 (t = -1.09)
DDD^d^ RR	0.78 [0.33; 1.80]	0.557 (t = -0.59)	0.39 [0.15; 1.01]	0.059 (t = -1.93)

^a^ >14 (women) and 21 (men) standard units of alcohol per week, or >5 standard units of alcohol per day

^b^Frequency of total abstinence in groups

^c^NDD: Number of drinking days

^d^DDD: Drinks per drinking day.

Moderate physical activity level was found to have a protective effect on drinking behavior compared to low physical activity levels among participants who completed the IPAQ at six months follow-up, including the controls. Participants with moderate level physical activity had lower odds for excessive drinking, OR = 0.12 [0.05; 0.31], p<0.001, and higher abstinence rate than participants with low level physical activity (Table 4).

**Table 4:** Effect of moderate level physical activity (PA) at follow-up in relation to low level physical activity at follow-up on AUD in total sample after six and 12 months in treatment.

Moderate level PA	Effect [95% CI]	p-value (test statistics), df
Excessive drinking^a^ OR	0.12 [0.05; 0.31]	p<0.001 (z = -4.399), 1
Abstinence rate^b^ OR	5.23 [2.19; 12.50]	p<0.001 (z = -3.725), 1

^a^ >14 (women) and 21 (men) standard units of alcohol per week, or >5 standard units of alcohol per day

^b^Frequency of total abstinence in groups.

### Dose-response

A dose-response effect of exercise was found. The amount of alcohol consumption in the intervention groups decreased by 4% [95% CI: 0.03–6.8%], p = 0.015 for each increased exercising day. That is, the more days participants recorded their exercise the less alcohol they consumed at six months follow-up.

## Discussion

To our knowledge, the present study is one of the largest RCTs to add an exercise intervention to treatment as usual for clinically diagnosed AUD. The intention-to-treat analysis revealed no statistically significant difference between intervention and control groups regarding alcohol intake measured at six months after treatment start. Our hypotheses on primary outcome of exercise as adjunctive treatment for AUD can therefore not be confirmed based on the primary analysis. Nor can our proposed secondary outcomes, as no difference in abstinence rates and number of drinks per drinking day was found between groups. However, our study demonstrated that a moderate level of general physical activity was associated with a lower probability of drinking behavior, and there was a significant dose-response effect of exercise on alcohol intake.

Only few studies have reported the effect of exercise on alcohol outcomes [5, 8, 13, 28] in terms of craving, abstinence rates and amount and frequency of alcohol use. We did not measure craving as an outcome. However, the studies by Sinyor et al. [28] and Brown et al. [5] both found a significant improvement in abstinence rates and reduced alcohol use measured three months post-intervention. Compared to these studies, our study had a longer follow-up period, and similarly to Brown et al. we found no effect of the intervention at the six months follow-up. The potential effect of adding exercise interventions to treatment as usual may therefore not lead to a lasting effect. The reason for this may be that participants’ adherence and motivation to exercise decreases in the months after initiation, e.g. due to the lack of an individually tailored exercise program or social support [29, 30], and this may explain why no intervention effect was observed at six months follow-up. Nevertheless, the dropout rate in the present study was 37.1%, which is lower than the 40% reported by Hallgren et al. [12] in their recent meta-analysis. Similarly, the most recent meta-analysis of exercise interventions for alcohol dependence showed no effect on drinking outcome, but significant and positive effects on other important health outcomes, such as fitness and depression [12]. Considering that individuals with alcohol dependence often have poor physical health and mental problems comorbid with alcohol use disorder, which can be improved by engaging in physical exercise [9], it is worth implementing *at least* advice on lifestyle change in existing treatment.

The general level of physical activity across groups was found to be associated with reduced alcohol intake. This indicates that, to some extent, participants in the control group also increased their physical activity level during the intervention period, despite not being instructed to do so. We may assume that all patients who accepted participation in the study had prepared themselves for the possibility of being randomized into a group including exercise; hence, all patients were probably somewhat interested in increasing their level of physical activity. Conversely, it is also possible that physical activity compensation may have occurred in one or both of the exercise groups. For example, people who started exercising may have done less physical activity outside the exercise intervention because they are now exercising regularly. Thus, their total physical activity may have stayed the same or even possibly declined. Furthermore, the finding supports the argument that a physically active lifestyle may play a supportive role in the treatment of AUD [31], as well as other types of substance use disorder (SUD), as former studies have also suggested [32]. Moreover, physical inactivity was found to be associated with an increased risk of alcohol dependence [33, 34], which further supports our argument that a moderate level of physical activity may play a protective role against AUD. Therefore, it may be a reasonable strategy to encourage physical activity in future AUD treatments. Furthermore, advice on reducing sedentary behavior and increasing light physical activity in everyday life can be addressed in future research, as it is suggested that there is a link between sedentary behavior and common mental disorders [34]. In the outpatient treatment clinic, motivational interviewing is already used, and physical activity could with advance be addressed during the sessions. Even a randomized study could be conducted testing the effect of motivational interviewing for enhanced physical activity for patients in treatment for AUD. It is thus strongly recommended to test the effect of physical activity on alcohol outcome in a study with a randomized controlled approach.

Patients in treatment for AUD may be considered as a group of individuals who are engaged in a behavior change process. Prochaska et al. [35] have reviewed the literature of multiple health behavior change research and found studies suggesting concurrent behavior change as well as studies showing no evidence of concurrent behavior change. It was suggested that change in one of the behaviors might support change in another. In continuation of this, a change in alcohol consumption may support change in other lifestyle factors, such as physical activity, or vice versa, which may explain why the participants in our present study increased their physical activity level. The opposite may also be the case; namely, that changing alcohol behavior may make such demands on personal resources that no further behavioral change can be expected from individuals in treatment for AUD [36].

This may explain why, along with possible motivational issues such as barriers to adherence, the present study saw a relatively high number of dropouts. Roessler et al. [37] argue that including motivational aspects of participation such as individually targeted exercise strategies or the involvement of their social network is important to enhance adherence. Some suggestions from Roessler et al. [37], regarding motivational aspects of participation include individual locus of control, self-efficacy, coping strategies and intrinsic motivation which should be considered in the design phase of an exercise intervention.

Our participants were randomized to either individual or group condition independent of their place of residence, work situation, or any psychosocial considerations. This might have given rise to a wide range of barriers for participation in exercise, as described in a qualitative study of dropouts in the Healthy Lifestyle Study [30]. Participating, for example, in group exercise with other alcohol patients requires personal strength or at least suitable preparation. In future interventions, we need therefore to focus more on transfer-oriented aspects, as, for example, the strengthening of relationships between patients during the intervention, supportive relations in their everyday life, or compliance in general [38].

Furthermore, we found that an increasing number of exercise days was associated with lower alcohol consumption among the participants in the intervention groups. This finding is interesting and demonstrates a positive relationship between exercise and outcome of AUD treatment. Naturally, this analysis did not include the control group as we did not ask them to record their exercise activity systematically during the intervention period. However, among individuals who were allocated to the intervention groups, alcohol consumption was reduced as exercising days increased. Brown et al. [5] also reported a dose-response effect of exercise. They found an inverse relationship between alcohol use and minutes of exercise. These associations can help future studies further investigate how much exercise (weekly/daily) is needed to obtain the best possible treatment outcome.

### Strengths and limitations

An important study limitation was the relatively high number of dropouts (37.1%), leaving the study unable to make sufficient measurements across the whole sample at follow-ups [30]. Furthermore, exercise activity was not recorded in the control group, so we can only assume or guess what happened in this group during treatment. Future studies should take this into consideration. In addition, the intervention should be more flexible, allowing participants to choose between different activities. It is possible that particularly the type or level of activity in the group exercise session was insufficient to induce change. A limitation of the current study is that data on exercise adherence are not reported, which impedes interpretation of the findings. A future study of exercise for AUD will present adherence data.

Measurement of alcohol intake by TLFB, and of physical activity level by IPAQ, is measurement by means of self-report. Although both instruments possess reasonable validity, there may have been cases of underreporting alcohol use and overestimating physical activity. In addition, the IPAQ does not provide an adequate measure of sedentary behavior, which limits our knowledge about this in the sample [39, 40]. Moreover, the sample consists of a group of patients with AUD who might have a degree of cognitive impairment in the form of declined working memory [41–43]. Therefore, it might have been difficult for them retrospectively to recall the correct amount of alcohol consumed and physical activity performed.

This study included a relatively high number of patients in treatment for AUD, making it the largest to date. All participants were consecutive, which enabled the study to follow the patient from treatment start and to use exercise as an adjunct to treatment as usual concurrently. The study setting was not controlled or fixed, as participants were recruited from an ordinary outpatient treatment center. This allowed the participants to continue with their normal daily lives during treatment, and made it possible to investigate the effectiveness of the exercise intervention. No adverse events were observed.

## Conclusion

Findings from the Healthy Lifestyle Study support existing evidence of physical exercise as adjunctive treatment for alcohol use disorder; it may be effective. Moderate level physical activity was found to be protective against excessive drinking at follow-up. Therefore, in aiming to obtain the best possible outcome of alcohol treatment, the focus should be more towards establishing physically active lifestyles rather than on intervening with leisure-time physical exercise. Furthermore, the dose-response effect of exercise on drinking outcome supports the need for implementing physically active lifestyles for patients in treatment. More research into how physical activity or exercise interventions can improve alcohol outcomes, just as in other areas, such as depression and quality of life, is needed to evaluate the benefits of supporting current AUD treatment with exercise as a non-pharmacological treatment.

## Supporting information

S1 Checklist (DOCX)

S1 Protocol (PDF)
