# Supplementary material for: Exercise as adjunctive treatment for alcohol use disorder: A randomized controlled trial
Source: PLoS One. 2017 Oct 19;12(10):e0186076. doi: 10.1371/journal.pone.0186076 (PMC5648142; doi:10.1371/journal.pone.0186076)
Supplement: S1 Protocol — (PDF) [file pone.0186076.s002.pdf]

Project HEALTHY LIFESTYLE

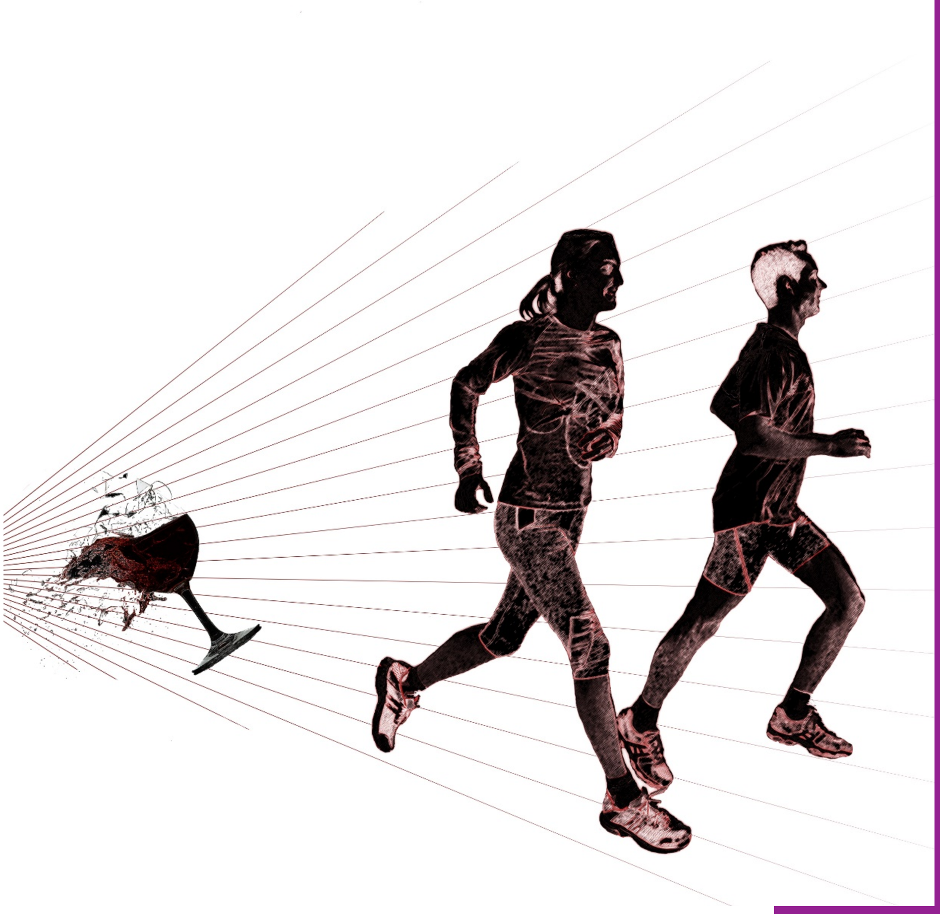

Physical exercise as a supplement to outpatient treatment of alcohol use disorders - a randomized controlled trial

Version 1.2

Project HEALTHY LIFESTYLE

Summary

Alcohol use disorder is a widespread problem in Denmark and has severe impacts on health and quality of life of each individual. The clinical treatment of alcohol use disorder involves evidence-based knowledge on medical treatment, physical training, and psychological management. The aim of this study is to investigate the effect of physical exercise on alcohol intake, cardio-respiratory fitness and socio-psychological outcomes.

The study is a randomized controlled trial with three arms: (A) Standard treatment alone, (B) Standard treatment + physical exercise in groups, or (C) Standard treatment + physical exercise on an individual basis. The patients will be interviewed and tested at baseline, and after 6 and 12 months.

We expect that alcohol consumption six months after start of treatment will be lowest in experimental group B (physical exercise in groups); a little bit higher in experimental group C (individual exercise) and highest in the control group A. We also expect compliance during treatment to be better in the experimental groups (B and C) than in the control group (A). Furthermore, we expect better quality of life, less anxiety and depression and better fitness in the experimental groups than in the control group.

Table of Contents

SUMMARY **2**

ADDRESSING THE CHALLENGE **4**

BACKGROUND AND RATIONALE **4**

PURPOSE AND HYPOTHESES **5**

MATERIALS AND METHODS **5**

STUDY DESIGN 5
PARTICIPATING PATIENTS 5
RANDOMIZATION AND BLINDING 6
INTERVENTIONS 6
PHYSICAL EXERCISE 7
EVALUATION INSTRUMENTS 8
OUTCOMES 9
Primary Outcome *9*
Secondary Outcome: *9*
SAMPLE SIZE AND STATISTICAL ANALYSES 10

ETHICS **11**

REFERENCES **12**

Addressing the challenge: A healthy lifestyle - it's not enough just to remove alcohol

The focus in treatment of alcohol use disorder needs widening. Physical exercise is known to produce health-related benefits for different target groups. In addition, it seems that physical exercise has a positive effect on physical, psychological and social consequences of alcohol abuse. Although exercise is a quite new treatment option to alcohol and substance abuse, the potential benefits of exercise for alcohol abusers are many and may reach beyond supporting the treatment of the alcohol dependence itself.

We propose **a study on healthy lifestyle** in order to improve the prognosis of patients with alcohol use disorder. The aim of the study is to test whether addition of moderate training to treatment for alcohol dependency will be more effective than alcohol treatment alone.

Background and rationale

In Denmark, alcohol leads to at least 3,000 potentially preventable deaths annually, representing 5.2% of total deaths. Furthermore, alcohol contributes to a large number of contacts with the health care system (Juel et al., 2006).

The vast majority of services offered to patients suffering from alcohol use disorders are publicly funded. Those who seek treatment represent dependent drinkers. The duration of the alcohol problem is on average ten years at the time of initial contact to the treatment system (Søgaard Nielsen et al, 2006).

Evidence-based treatment of alcohol dependence includes different psychological interventions and pharmacological treatment (National Institute for Health & Clinical Excellence, 2011). The outcome of current alcohol treatment is modest (Cutler & Fishbain, 2005); relapse in the first year after treatment ranges between 60 and 90% (Miller et al., 2001). There is a strong need for developing interventions that can increase the effectiveness of treatment.

Methods that foster healthy lifestyle changes are likely to contribute to the long-term maintenance of recovery of alcohol abuse. Interventions targeting physical activity in particular, may be especially valuable as adjunct to alcohol treatment.

Physical exercise is known to produce health-related benefits for different target groups (Pedersen & Saltin, 2006), for example improved fitness or weight control (Chaput et al., 2011; Cornellisen & Fagard, 2005). For substance abuse, exercise is a quite new and promising treatment option (Moore et al., 2005). Physical exercise can be used both as early prevention, and as part of a continuous treatment process (Collingwood et al., 2005; Biddle & Mutrie, 2005).

With regard to alcohol abusers, several mechanisms can be pointed out. Exercise, especially moderate exercise (Monti et al., 2000), can decrease the *urge* to drink. Exercise may offer positive alternatives to alcohol by triggering *pleasurable states*, for example through *dopaminergic reinforcement* (Read & Brown, 2003). Exercise also improves psychosocial outcomes in the areas of *mood management* (Lane & Lovejoy, 2005) and reduces

depression and *anxiety* (Martinsen, 2008; Babyak et al., 2000; DiLorenzo et al., 1999). In addition, resilience factors such as individual and *social resources* (for example self-confidence) are strengthened by regular physical activity, especially as group activity (Brown et al., 2009; Read & Brown, 2003).

Despite the potential benefits of exercise interventions, only few studies have tested the impact of exercise as an adjunct to alcohol treatment (Trivedi et al., 2011; Murphy et al., 1986; Sinyor et al., 1982; Brown et al., 2009). Findings from the studies support a positive relationship between physical exercise and drinking outcome. However, most of the studies suffer from methodological limitations such as small sample sizes or high dropout.

The overall purpose of the present study is to evaluate the effect of adding exercise to treatment of outpatients with alcohol use disorder.

Purpose and hypotheses

The specific objectives of this study are to examine whether physical activity done alone or in groups as an adjunct to outpatient alcohol treatment has an effect on:

1. Alcohol intake 6 months and 12 months after initiation treatment

2. Patients wellbeing, fitness, anxiety, depression and interpersonal problems

We hypothesize that physical exercise with moderate intensity yields significant clinical improvements.

Materials and methods

Study design

The study is a randomized controlled study with three arms: (A) Patients allocated to treatment as usual (B) Patients allocated to treatment as usual + physical exercise in groups, and (C) Patients allocated to treatment as usual + individual exercise

Participating patients

300 consecutive patients entering the alcohol outpatient clinic in Odense and suffering from alcohol use disorder: abuse or dependence according to DSM-IV-TR are enrolled in the study if they meet the following inclusion criteria:

1. Age between 18 and 60 years

2. Native Danish speaking

3. Have no severe psychosis or cognitive impairment

4. Have no severe physical disabilities or medical problems

5. Accept participating in the study

All new patients who start psychosocial treatment at the alcohol outpatient clinic in Odense will be referred to a research assistant, who will give oral and written information about the study.

If the research assistant has any doubt whether the patients fulfil the inclusion criteria, he will refer them to one of the outpatient clinics psychiatrists who – according to a clinical evaluation – will decide if they fulfil the inclusion and exclusion criteria. Patients who refuse to participate in

the study will not be asked to give a reason. The patients are informed, before the meeting to take a family member by the information meeting about the project.

A case report form for each participant will be prepared, labelled only with the participant number.

Figure 1. Study design

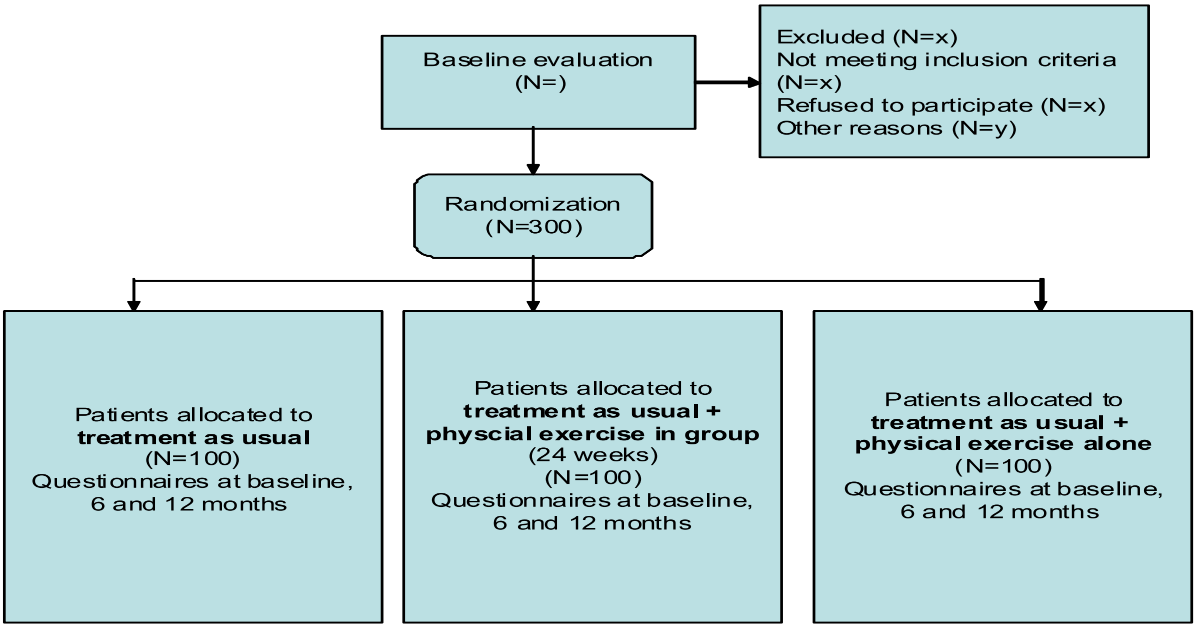

If the patients wish to participate, the research assistant offers additional information material about the study and the testing (see information material). After the patients have provided a written and an oral consent the baseline interview is carried out. Then the patients are randomized to (A) Treatment as usual (B) Treatment as usual + physical exercise in groups, or (C) Treatment as usual + physical exercise alone (Figure 1).

Randomization

Patients are randomized by block randomization by the Institute of Public Health, University of Southern Denmark.
The treatment staff is not blinded to which intervention the patients receive.

Consent and identification list with number will be stored in a locked cabinet out of reach for the research group. The two intervention groups will be coded, for example, “x” and “y”, throughout the analysis phase and when drawing the conclusions.

Interventions

Treatment as usual (TAU): All patients will receive the normal outpatient treatment for alcohol use disorder at the clinic. Treatment is carried out by an interdisciplinary team of nurses, psychiatrists and social work professionals (Nielsen et al., 2000). On submission, the patient may receive treatment for withdrawal symptoms. Subsequently, a screening interview is carried out by the clinical staff, using the Addiction Severity Index (ASI) (McLellan et al., 1980).

The attached psychiatrists make assignment to the individual treatment offer. The assignment is based on results of ASI and the psychiatrists' experience as to which patients will benefit the most from the various treatment offers.
Current treatment offers include family therapy, cognitive behavioural therapy, contract therapy and supportive consultations. After psychiatric evaluation, the patient may be offered pharmacological treatment consisting of Disulfiram, Naltrexone, Acamprosat or antidepressant medication.

The duration of the TAU and the frequency of sessions follow the usual guidelines for outpatient alcohol treatment in Denmark. The therapists are well educated and have received training in the treatment methods that they offer. Frequent staff supervision takes place. For all treatment modalities, clinical guidelines are available.

Physical exercise

Patients in the two intervention groups will receive physical exercise + the outpatient treatment as described above (treatment as usual). The exercise training will be accomplished in cooperation with the Institute of Sports Science and Clinical Biomechanics in cooperation with the Department of Psychology at the University of Southern Denmark.

The distinction into individual and group exercise is chosen in order to investigate compliance to the treatment. The heart-rate monitors with USB sticks allow for every patient to transmit running distance and time directly to the computer system.

Patients in the intervention groups will follow a 24-week programme, either alone or in a training group (Table 1). The exercise involves brisk walking or running. After a ramp-up period of two weeks with only 30 minutes training sessions to minimize the risk of injury, the exercise sessions increases to two one hour exercise training sessions per week for a period of ten weeks. The walking or running ramp-up period consists of 25 min brisk walking including a number of 30 s running intervals all depending on individual fitness level. The duration of the running or walking intervals increases each week as the participants improve their fitness level. The intensity also increases to reach 45 min with 3-5 min running/brisk walking intervals (Heart Rate (HR) increase by 80–90 %) and 1 min rest (moderate walking HR increase by 50–60 %). All patients are requested to use heart rate monitors during exercising with USB sticks to monitor, measure and transmit heart rate and running distance directly into the computer system.

Table 1 Oversight of training schedules

	Individual	Group
Supervised instruction	4 sessions à 60 min:<br><br>2 sessions prior to start (providing running instructions & exercise program)<br>2 sessions following up (providing regulation of program)	36 sessions à 60 min:<br><br>2 weekly sessions during 12 weeks, followed by 1 weekly session during 12 weeks (providing running instructions & exercise program)

Exercise alone

Patients will receive an individual program and running instructions during two individual sessions prior to start after first testing. These sessions will be followed up by two more sessions, after 4 weeks and 12 weeks, respectively.

Exercise in groups
Patients will receive an individual program and running instructions depending on their level of experience. They will exercise in a group with an exercise frequency during the first 12 weeks of 2 hours per week (including a ramp-up period of two weeks), followed by 12 weeks with a supervised training frequency of one hour per week. The patients are asked to exercise at least twice a week and to continue on their own upon completion of the supervised exercise programme.

Exercise safety
Prior to each exercise session, participants will sit undisturbed for 5 minutes before assessment of their resting HR and blood pressure (BP). If a participant's resting HR is ≥ 100, it will be re-measured after an additional 5-minute rest period. If a participant's resting HR remains ≥ 100, the exercise session will be rescheduled for another day. Likewise, if a participant's resting BP is ≥ 160/100, it will be re-measured after an additional 5-minute rest period. If a participant's resting BP remains ≥ 160/100, the exercise session will be rescheduled for another day. Guidelines are also presented for referral to appropriate medical care and additional physician clearance based on blood pressure readings.

Evaluation instruments

Evaluation instruments applied in the study are:
The Addiction Severity Index (ASI). ASI provides a multidimensional image of the patient's situation within the last month before the interview. The interview concentrates on the following seven areas in the patient's life: medicine, employment, alcohol, drug, legal status, family/social network, and psychiatric health. ASI contains two different scores: the interviewer score (self-reported estimation of the interviewer) and the composite score (self-reported estimation of the interviewer). In the study only the composite score will be used. The scores give a mathematical estimate of each problem area based on symptoms within the 30-day period preceding the interview. Each composite score consists of the sum of various questions from the ASI. Final scores are reported on a decimalscale as 0 to 1, where 0 denotes no problems and 1 denotes severe problems with alcohol.

The time-line-follow-back method *(TLFB)* is used to describe alcohol-free days as well as number of drinks per day. By use of TLFB patients describe the daily number of standard drinks 30 days before the basic interview and 30 days before the 6 and 12-month follow-up interview.

Cardiorespiratory fitness: VO_{2max} is assessed using the Bruce treadmill test (Bruce et al., 1963) with increased speed (2.7 km/h, 5.5 km/h, 6.8 km/h) and grade (10%, 12%, 14%, 16%) every 3 minutes according to a scheme. Oxygen consumption is measured directly by the breath. The test continues until exhaustion.
To avoid discomfort with the testing standardised written and oral information is applied.

Physical activity: 1) Prior to treatment: The level of physical activity is assessed using the International Physical Activity Questionnaire (IPAQ), a 27-item self-completion questionnaire. It measures activities taken in

each of the four domains: leisure-time physical activity; domestic and gardening activities; work-related physical activity and transport-related physical activity.
2) During treatment: the physical activity will be measured by HR monitors with possibility for valid registration of activity.

Well-being is assessed by EuroQuol-5D (EQ-5D), a standardized instrument for use as a measure of health outcome, functioning and health status.

Anxiety and depression: is assessed by the Screening Questionnaire of Common Mental Disorders (CMD-SQ) consisting of 37 items in validated subscales (SCL-SOM, Whiteley-7, SCL-ANX-4, SCL-8, SCL-DEF-6, CAGE) measuring anxiety, depression, use of alcohol, and somatisation. The patients respond on a five point Likert scale. A normal score is <= 4 in somatisation (SCL-SOM) and 0 in the other scales.

Interpersonal problems: are assessed by the IIP (Inventory of Interpersonal Problems). The measurement of interpersonal problems allows a differentiation of interpersonal and non-interpersonal sources of distress (e.g. depressed mood, anxiety). The IIP (short form) consists of 64 items scored on eight scales. The scales include areas that may be hard for a person and areas that indicate things a person may do too much. The eight scales (domineering, vindictive, cold, socially avoidant, non-assertive, exploitable, overly nurturant and intrusive) are scored on at five point scale.

At the 12-month follow-up interview, information regarding treatment is recorded – in addition to the evaluation instruments mentioned above. Disclosure of case notes describes number of treatment sessions, discontinuation of treatment and treatment period.

Outcomes

Primary Outcome

The primary endpoint analysis (6 months) will be a comparison of outcomes for patients assigned to TAU (A) versus the combined physical exercise experimental groups TAU + group exercise (B) and TAU + individual exercise (C). The outcome will be measured by the proportion of patients with sensible drinking according to the limits by the Danish National Board of Health (Sensible drinking is defined as drinking maximum 14/21 drinks/week among women/men, one drink contain 12 grams of pure alcohol). The primary outcome will be in the intention to treat group using last observation carried forward.

Secondary Outcome

1. 12 months analysis
2. The health status
3. The per cent of patient with reduced depression, anxiety and interpersonal problems
4. Maximum oxygen uptake

For each outcome goal, two analyses will be carried out:

1. Intention-to-treat analyses will be carried out for all patients, irrespective of whether they completed the interventions or were re-interviewed. With regard to incomplete data, "last observation carried forward" and multiple imputations will be used.
2. Completer (on-treatment) analyses will be carried out for patients who

completed the interventions. This sentence is unclear

3. In addition, non-completer (on-treatment) analysis will be carried out by interviews.

Sample size and statistical analyses

To our knowledge no similar studies have been conducted. Therefore, the power calculation is estimated from quality assurance data and research data of the participating alcohol clinic in Odense. Currently 65 % of the patients have sensible drinking 6 months after starting treatment with the current treatment regime (Data from alkoholambulatoriet i Odense, 2011).

Figure 2. Sample size calculation

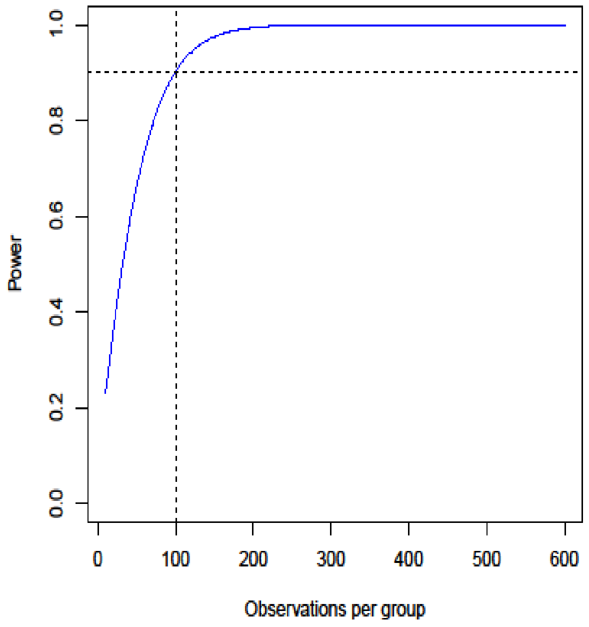

In this study we compare both TAU (A) with TAU + group running (B) and TAU (A) versus TAU + individual running (C). A sample of 100 patients in each group is needed to have 90% power of detecting a difference corresponding to an improvement of 18 percentage points using a 5% level of statistical significance (figure 2). Since the two primary endpoints are the comparison of each of the additional exercise groups to the regular TAU treatment, the sample size is relevant for all three treatment arms. Should the data subsequently show that individual and group exercises are comparable then the total power will be increased.

All the participants will be measured by questionnaires and physical activity at baseline, and 6 and 12 months from baseline.

The data will be analysed by a logistic regression model to model the proportion of patients with sensible drinking. The logistic regression modelling allows for inclusion of additional confounders. A backward elimination strategy will be employed to identify significant explanatory variables, using a significance level of 0.05. Generally, two-sided alternative will be considered except when comparing TAU + physical exercise to TAU without physical exercise, where a one-side alternative is used. Explanatory variables considered will include age, gender, as well as other relevant variables available.

Budget

Professor Kirsten Kaya Roessler has taken the initiative to this project and is the head chef of the project.

The project has be a part of a common application of five studies. All five studies have got 63 million Danish kroner from the Lundbeck foundation, the Danish foundation Trykfonden and the Region of the Southern Denmark. This project has got the 4.3 million from the Danish foundation, Trykfonden.

There are no cooperation between the head chef of this project and the Danish

foundation, Trykfonden. The money is received.

The patients are not getting any money if they participate in the project or not.

Ethics

All patients will receive conventional baseline treatment for their alcohol use disorder. We do not know whether the addition of psychical exercise will improve the effect of the treatment for alcohol use disorders, and since treatment for alcohol use disorders without addition of an exercise programme is the standard treatment of today, we find no ethical problems in the study.

In the intervention group, the exercise programme is based on moderate exercise and, hence, can be performed by all participants. Any patient not wishing to participate in the study will be treated for alcohol use disorder as usual, that is without the addition of physical exercise.

All data collected in the study will be treated strictly confidentially. No analysis or publication will contain information that allows person identification. Before initiation of the study, the protocol will be approved by the Regional Scientific Ethical Committee for Southern Denmark and the Danish Data Protection Agency. All procedures in the study are in accordance with the second Declaration of Helsinki.

The publications

The results both positive and negative will be publicised in international journals and present on relevant conferences.

References

Babyak, M., Blumenthal, J.A., Herman, S., Khatri, P., Doraiswamy, M., Moore, K., Craighead, E., et al. (2000). Exercise treatment for major depressions: maintenance of therapeutic benefit at 10 months. Psychosomatic Medicine, 62, 633–638.

Biddle, S. & Mutrie, N. (2005). Psychology of physical activity: Determinants, Well-being and Interventions. London: Routledge.

Brown, R.A., Abrantes, A.M., Read, J.P., Marcus, B.H., Jakicic, J., Strong, D.R., Oakley, J.R., Ramsey, S.E., Kahler, C.W., Stuart, G., Dubreuil, M.E. & Gordon, A.A. (2009). Aerobic exercise for alcohol recovery: Rationale, program description, and preliminary findings. Behavior Modification, 33(2), 220-249.

Chaput, J.P., Klingenberg, L., Rosenkilde, M., et al. (2011). Physical Activity Plays an Important Role in Body Weight Regulation. Journal of Obesity.doi:10.1155/2011/360257

Collingwood TR, Sunderlin J, Reynolds R, Kohl HW. Physical training as a substance abuse prevention intervention for youth. J Drug Educ 2000; 30(4):435–51.

Cornellisen, V. A. & Fagard, R. H. (2005). Effects of endurance training on blood pressure, blood pressure-regulating mechanisms, and cardiovascular risk factors. Hypertension, 46, 667–675.

Cutler RB, Fishbain DA. Are alcoholism treatments effective? The Project MATCH data. BMC Public Health 2005; 5:75.:75.

DiLorenzo, T.M., Bargman, E.P., Stucky-Ropp, R., Brassington, G.S., Frensch, P.A. & LaFontaine, T. (1999). Long-term effects of aerobic exercise on psychological outcomes. Preventive Medicine, 28, 75–85.

Juel, K., Sørensen, J., Brønnum-Hansen, H. (2006). Risikofaktorer og folkesundhed I Danmark. København: Statens Institut for **F**olkesundhed.

Lane, A.M. & Lovejoy, D.J. (2005). The effects of exercise on mood changes: the moderating effect of depressed mood. Journal of Sports Medicine and physical fitness 41, 4: 539-548.

Martinsen, E.W. (2008) Physical activity in the prevention and treatment of anxiety and depression. Nord J Psychiatry 62, Suppl 47:25-29.

McLellan, A.T., Luborsky, L., Woody, G.E., O'Brien, C.P. (1980). An improved diagnostic evaluation instrument for substance abuse patients. The Addiction Severity Index. *J Nerv Ment Dis*; 168(1): 26-33

Miller, W.R., Willbourne, P.L. (2002). Mesa Grande: a methodological analysis of clinical trials of treatments for alcohol use disorders. Addiction; 97:265-277.

Moore, M.J., Werch, C.E.C. (2005). Sport and physical activity participation and substance use among adolescents. J Adol Health; 36(6):486–93.

Monti, P.M., Rohsenow, D.J., & Hutchison, K.E. (2000). Toward bridging the gap between biological, psychobiological and psychosocial models of alcohol craving. Addiction 95 (Suppl 2), 229-236.

Murphy, T.J., Pagano, R.R., Marlatt, A. (1986). Lifestyle modification with heavy alcohol drinkers: Effects of Aerobic and meditation. Addictive Behaviors; 11:175-186.

National Institute for Health & Excellence (2001). Alcohol-use disorders. Diagnosis, assessment and management of harmful drinking and alcohol dependence. National Clinical Practice Guideline 115. London.

Nielsen, B., Nielsen, A.S., Wraae, O. (2000). Factors associated with compliance of alcoholics in outpatient treatment. J Nerv Ment Dis: 188(2): 101-7.

Nielsen B., Nielsen A.S. (2001). Odensemodellen – Et bidrag til en evidensbaseret praksis ved behandling af alkoholmisbrugere. ISBN 87 7343 4817.

Pedersen, B.K. & Saltin, B. (2006). Evidence for prescribing exercise as therapy in chronic disease. Scandinavian Journal of Medicine and Science in Sports, 16 Suppl. 1, 3–63.

Read, J.P. & Brown, R.A. (2003). The Role ofPhysical Exercise in Alcohol Treatment and Recovery. Professional Psychology: Research and Practice.

Sinyor D, Brown T, Rostant L, Seraganian P (1982). The role of Physical Fitness Program in the treatment of alcoholics. J Stud Alco;43:380-86.

Søgaard Nielsen, A., Becker, U., Højgaard, B., Lassen, A.B., Willemann, M., Søgaard, J., Grønbæk, M. (2006). Alkoholbehandling – en medicinsk teknologivurdering. Center for Evaluering og Medicinsk Teknologivurdering. Medicinsk Teknologivurdering; 8(2). København: Danish National Board of Health;.

Trivedi, M. et al. (2011). Stimulant Reduction Intervention using Dosed Exercise (STRIDE) - CTN 0037: Study protocol for a randomized controlled trial. Trials. 2011; 12: 206

Project HEALTHY LIFESTYLE

Version 1.2
